# Multispectral-derived genotypic similarities from budget cameras allow grain yield prediction and genomic selection augmentation in single and multi-environment scenarios in spring wheat

**DOI:** 10.1007/s11032-024-01449-w

**Published:** 2024-01-15

**Authors:** Tomasz Mróz, Sahameh Shafiee, Jose Crossa, Osval A. Montesinos-Lopez, Morten Lillemo

**Affiliations:** 1 Department of Plant Sciences, Norwegian University of Life Sciences, NO-1432 Ås, Norway; 2 International Maize and Wheat Improvement Center (CIMMYT), Km 45, Carretera Mexico Veracruz, CP 52640 Texcoco, Edo. de Mexico Mexico; 3 Colegio de Postgraduados, CP 56230 Montecillos, Edo. de Mexico Mexico; 4 Facultad de Telemática, Universidad de Colima, Colima, Mexico

**Keywords:** Spring wheat, Grain yield, Grain yield prediction, Genomic prediction, Multispectral imaging, High-throughput phenotyping

## Abstract

With abundant available genomic data, genomic selection has become routine in many plant breeding programs. Multispectral data captured by UAVs showed potential for grain yield (GY) prediction in many plant species using machine learning; however, the possibilities of utilizing this data to augment genomic prediction models still need to be explored. We collected high-throughput phenotyping (HTP) multispectral data in a genotyped multi-environment large-scale field trial using two cost-effective cameras to fill this gap. We tested back to back the prediction ability of GY prediction models, including genomic (G matrix), multispectral-derived (M matrix), and environmental (E matrix) relationships using best linear unbiased predictor (BLUP) methodology in single and multi-environment scenarios. We discovered that M allows for GY prediction comparable to the G matrix and that models using both G and M matrices show superior accuracies and errors compared with G or M alone, both in single and multi-environment scenarios. We showed that the M matrix is not entirely environment-specific, and the genotypic relationships become more robust with more data capture sessions over the season. We discovered that the optimal time for data capture occurs during grain filling and that camera bands with the highest heritability are important for GY prediction using the M matrix. We showcased that GY prediction can be performed using only an RGB camera, and even a single data capture session can yield valuable data for GY prediction. This study contributes to a better understanding of multispectral data and its relationships. It provides a flexible framework for improving GS protocols without significant investments or software customization.

**Supplementary Information:**

The online version contains supplementary material available at 10.1007/s11032-024-01449-w.

## Introduction

To develop new plant varieties and cultivars, breeders initially relied solely on recorded phenotypes of candidates paired with “the breeder’s eye.” With increasing pressure caused by the climate change, increasing world population, and diminishing arable land, developing new and adapted germplasm is more urgent than ever (Hickey et al. 2019). Nowadays, plant breeders have an abundance of new and innovative tools at their disposal to aid their quest for better-adapted germplasm, focusing on selection accuracy, breeding cycle shortening, and maximizing the genetic pool to be screened—therefore effectively accelerating genetic gains in all aspects of the breeder’s equation (Houchmandzadeh 2014).

Genomic selection (GS), first proposed by Meuwissen et al. (2001), aims to estimate breeding values (GEBVs) of individuals that have been genotyped-but not phenotyped—based on prediction equations developed from a collection of phenotyped and genotyped individuals. New parents for crossing are then selected based on the GEBVs, which shortens the breeding cycle since late filial generations do not need to be phenotyped for quantitative traits such as grain yield (GY) (Bassi et al. 2015). Due to the cost reduction of genotyping and well-elaborated methodologies, GS has become routine in many breeding programs (Bhat et al. 2016).

With abundant genomic data, plant phenotype registering became a bottleneck in plant research and breeding, stimulating the development of high-throughput phenotyping (HTP) methodologies. HTP involves automating the evaluation of plant phenotypes and was enabled by recent advancements and the popularization of sensor and computing technologies paired with data analytics (White et al. 2012); it allows to cover large numbers of genotypes in a fraction of the time needed for manual measurements (Araus & Cairns 2014; Burud et al. 2017). HTP has shown considerable potential by enabling GY prediction using machine learning, as reviewed by van Klompenburg et al. (2020). HTP data has proven useful also in predicting above-ground biomass (Han et al. 2019; Lu et al. 2019; Li et al. 2020), plant height (Hu et al. 2018; Hassan et al. 2019; Tirado et al. 2020), earliness (Zhou et al. 2019; Trevisan et al. 2020), and crop emergence (Li et al. 2019) to name just a few.

A specific branch of HTP uses unmanned aerial vehicles (UAVs) equipped with multispectral or hyperspectral cameras, which record light spectrum above and beyond the visible spectrum. The usefulness of recording wavelengths outside the visible spectrum lies in their link with various aspects of crop physiology or chemistry. For instance, near-infrared (NIR, 760–1400 nm) is linked to crop water status; RedEdge (around 730 nm) is arguably a proxy of chlorophyll content (Peñuelas & Filella 1998); and ultra-violet A (UV-A, 200–380 nm) can be used to monitor stress in plants (Brugger et al. 2019). This extra information can help to construct vegetation indices (VIs), which are linear combinations of reflectance values such as NDVI (normalized difference vegetation index, Beisel et al. 2018) and, in turn, can be used for primary trait prediction (Montesinos-López et al. 2017; Shafiee et al. 2021).

HTP data gathered using multispectral and hyperspectral cameras has also been used to improve the accuracy of GS, as first demonstrated by Rutkoski et al. (2016), where secondary VIs increased GY prediction accuracy by 70%. HTP can help measure genetically correlated secondary traits, which can be introduced into multivariate prediction models (Sun et al. 2017; Sakurai et al. 2022). Likewise, HTP data was also discovered to help evaluate genetic resources for the expression of complex traits (Reynolds & Langridge 2016). In a recent study, NIR spectra of grain samples were used to construct spectral relationship matrices to enable phenomic selection (PS) and to aid GS, showing that the hyperspectral matrix-aided best linear unbiased prediction (H-BLUP) model performed at least as well as the standard genomic best linear unbiased prediction (G-BLUP) model. A model combining both spectral and genomic information (GH-BLUP) was superior to both G and H-BLUP alone (Robert et al. 2022a), showing similar results to Krause et al. (2019). Time-series drone multispectral data allowed also for tree growth parameter prediction in slash pine using a linear kernel constructed based on vegetation indexes and band values (Li et al. 2023). The PS based on the NIR spectra was also a promising, low-cost alternative to genotyping and a viable approach for predicting complex traits in perennial species such as grapevine (Brault et al. 2022). NIR spectra are usually acquired in most breeding programs for seed composition estimation and are therefore available without additional costs. However, their usefulness in predicting seemingly unrelated traits like GY must be questioned (Dallinger et al. 2023). To the authors’ knowledge, no attempt has been made to utilize genetic relationships derived from low-cost multispectral imagery for GY prediction in wheat and augmenting GS protocols.

To fill this gap, we deployed HTP in a multi-environment spring wheat trial using two cost-effective multispectral cameras mounted on commercial UAVs. We tested various back-to-back GY prediction models using genomic (G) and multispectral (M) relationships combined with environment-specific phenotypical covariates. We investigated the applicability and flexibility of environment-specific M relationships in single and multi-environment scenarios and their synergy with the GS-GBLUP model. As such, the main objectives of this study were to.

1. Investigate the prediction ability of multispectral-derived genetic relationships for GY in single and multi-environment scenarios.
2. Verify the possibility of augmenting GS with multispectral-derived genetic relationships.
3. Study which multispectral band(s) are the most important for GY prediction.
4. Examine the most informative data capture time for GY prediction under Norwegian growing conditions.

## Materials and methods

### Plant material

The Norwegian University of Life Sciences (NMBU) spring wheat panel, consisting of 301 hexaploid spring wheat (*Triticum aestivum* L.) cultivars and breeding lines, was used for the study. The same panel was recently used for genetic analyses of GY (Mróz et al. 2023), Fusarium head blight (Nannuru et al. 2022), and *Septoria nodorum* blotch (Lin et al. 2022) resistance. The collection encompasses 186 Norwegian, 40 Swedish, and 37 lines from CIMMYT, with several additional lines from Australia, Brazil, Canada, Czech Republic, Denmark, Finland, France, Germany, Netherlands, Poland, Russia, Slovakia, South Africa, Switzerland, UK, and the USA. The whole set presents a broad genetic and phenotypic diversity.

### Field trials

Trials were carried out during field seasons 2015–2022 between April and August in Vollebekk Research Station (Norway, Ås, 59° 39′ N, 10° 45′ E) and Staur Farm (Norway, Stange, 60° 43′ N, 11° 06′ E), which represent the two principal economically important wheat-growing areas in Norway due to the somewhat warmer and milder climate of south-eastern Norway and the slightly colder and temperate climate of inland Norway.

The trials were fertilized at sowing with 120 kg∙ha^−1^ of compound NPK fertilizer (YaraMila 22–3-10) and planted each season in both locations in late April or early May (exact planting dates in Table S1). Following germination, trials were kept disease- and weed-free according to local management practices using herbicides (Tripali [active ingredients: florasulam + metsulfuron-methyl + tribenuron-methyl] and Duplosan Meko [mekoprop]) and fungicides (Proline [prothioconazole], Aviator Xpro [bixafen + prothioconazole], Forbel [fenpropimorph] and Comet Pro [pyraclostrobin]) in doses tailored to the needs. Irrigation was applied in case of drought that could affect the growth of the plants. Alleys within the trials were created by spraying glyphosate shortly after seedling emergence. The trials were harvested each season towards the end of August after all varieties had reached full ripeness.

### Field trial design

The trials were designed as an alpha-lattice with two replicates per genotype and a block size of 6 with positions of every accession randomized each year. Each column was planted with buffer variety at its start and end to eliminate border effects. Each field trial plot was 5 × 1.5 m in size at harvest, with gaps between the plots of 30 cm and a central alley of 1 m. For the main panel, not every variety was tested in each year/location, and the number of genotypes tested varied from 100 to 295 per year/location.

### GY and phenology data

GY was measured in two locations over seven field seasons (a total of 11 environments–year/location combinations): Vollebekk Research Station in 2015, 2016, 2017, 2019, 2020, 2021, and 2022; Staur Farm: 2016, 2017, 2019, and 2020.

GY was measured by harvesting and threshing the trial plots, drying the yield until approximately 13.5% moisture, weighing it, and recalculating it to grams per square meter. The occurrence of two phenological stages (heading and maturity) was also recorded in each environment to localize the flight missions in the growing season. Heading was defined as the moment when approximately 50% of the tillers unveiled their heads. Maturity was assessed based on discoloration and ripening of peduncles and was defined as the moment when approximately 50% of the peduncles were ripe.

Data for plots lodged early was removed due to the heavy impact on their development. If lodging occurred late in the season (close to maturity), data were double-checked for consistency and possible impact on the traits.

### Statistical analysis of the field trial data

For GY, three types of adjusted genotypic means (BLUEs, best linear unbiased estimators) were calculated: year/location (environment) mean, location mean (all years from one location), and a global mean (where all the environments were combined).

As it was not uncommon to observe extra spatial variability within the trials (due to soil gradients) that was not fully captured by blocking, an additional covariate was introduced (columns) into the models to correct for it. The BLUEs were calculated using packages “lme4” and “lmerTEST” and custom scripts in R, version 4.2.1 (R Core Team 2021).

Environment (field trial) BLUEs were calculated using the mixed model (1):

1 $${P}_{ilmn}=\mu +{g}_{i}+{R}_{l}+{R:B}_{lm}+{C}_{n}+{e}_{ilmn}$$

where ${P}_{ilmn}$ denotes the response variable measured in the $i$ th genotype, $l$ th replication, $m$ th block and $n$ th column; $\mu$ denotes a general mean or intercept; ${g}_{i}$ denotes the fixed effect of genotype $i$, with $i=1,\dots , I$; ${R}_{l}$ denotes the random effect of replication effect; ${R:B}_{lm}$ denotes the random effect of block $m$ nested in replication $l$; ${C}_{n}$ denotes the random effect of column effect; and ${e}_{ilmn}$ is the error random term. All random effects are assumed to be normally distributed with zero mean and the respective variances.

BLUEs for each location were calculated using the mixed model (2):

2 $${P}_{iklmn}=\mu +{g}_{i}+{Y}_{k}+{Y:R}_{kl}+{Y:R:B}_{klm}+{Y:C}_{kn}+{e}_{iklmn}$$

where ${P}_{iklmn}$ denotes the response variable measured in the $i$ th genotype, $k$ th year, $l$ th replication, $m$ th block, and $n$ th column. ${Y}_{k}$ denotes the random effect of year effect, ${Y:R}_{kl}$ denotes the random effect of replication $l$ nested in year $k$, ${Y:R:B}_{klm}$ denotes the random effect of block $m$ nested in replication $l$ nested in year, ${Y:C}_{kn}$ denotes the random effect of column $n$ nested in year $k$, and ${e}_{iklmn}$ is the error term. All random effects are assumed to be normally distributed with zero mean and the respective variances.

Global BLUEs (cross-year, cross-location) were calculated using the mixed model (3):

3 $$\begin{array}{c}{P}_{ijklmn}=\mu +{g}_{i}+{L}_{j}+{Y:L}_{jk}+{Y:L:R}_{jkl}+{Y:L:R:B}_{jklm}+{Y:L:C}_{jkn}+{e}_{ijklmn}\end{array}$$

where ${P}_{ijklmn}$ denotes the response variable measured in the $i$ th genotype, $j$ th location, $k$ th year, $l$ th replication, $m$ th block, and $n$ th column. ${L}_{j}$ denotes the random effect of location; ${Y:L}_{jk}$ denotes the random effect of location $j$ nested in year $k$; ${Y:L:R}_{jkl}$ denotes the random effect of replication $l$ nested in location $j$ nested in year $k$; ${Y:L:R:B}_{jklm}$ denotes the random effect of block $m$, nested in replication $l$ nested in location $j$ nested in year $k$; ${Y:L:C}_{jkn}$ denotes the random effect of column $n$ nested in location $j$ nested in year $k$; and ${e}_{ijklmn}$ is the random error term. All random effects are assumed to be normally distributed with zero mean and the respective variances.

In the single-environment scenario (“Model performance assessment”), environment, location, and global BLUEs were used. In the multi-environment scenario (“Model performance assessment”), only environment BLUEs were used. Broad-sense heritability (*H*^2^) was calculated for individual trials using Eq. (4) (Falconer & Mackay 1996):

4 $${H}^{2}=\frac{{\sigma }_{G}^{2}}{{\sigma }_{G}^{2}+ \frac{{\sigma }_{e}^{2}}{r}}$$

where ${\sigma }_{g}^{2}$ is the genotypic variance, *r* is the number of replicates, and ${\sigma }_{e}^{2}$ is the error variance. Variance components for Eq. (4) were estimated using package “lme4” using the just described models but assuming the lines (genotypes) as normally distributed with mean zero and variance ${\sigma }_{G}^{2}$.

### Genotyping data

Samples were prepared and genotyped as described in Nannuru et al. (2022).

Physical positions of the markers were determined using the chip’s documentation, and markers which were not mapped to any physical chromosome position were placed on a fictional chromosome Un.

Markers were filtered, leaving only those with less than 10% missing data and minor allele frequency (MAF) larger than 0.05. Heterozygous markers were treated as missing data. After the quality check, the dataset contained 19,874 high-quality markers mapped to sub-genomes A (7999), B (7905), and D (2111) on chromosomes 1A (1156), 1B (1147), 1D (391), 2A (1232), 2B (1377), 2D (437) 3A (1074), 3B (1336), 3D (256), 4A (699), 4B (602), 4D (111), 5A (1340), 5B (1406), 5D (311), 6A (1126), 6B (1082), 6D (319), 7A (1372), 7B (955), 7D (285), and Un (1859).

### High-throughput phenotyping data

High-throughput phenotyping data were captured using two cameras: Micasense RedEdge M (https://micasense.com) and DJI Phantom 4 Multispectral camera (https://www.dji.com/p4-multispectral). In both locations, the RedEdge M camera was used during field seasons 2019–2021, whereas the Phantom 4 Multispectral was used during field season 2021 in Vollebekk Research Farm.

Detailed UAV specifications and the HTP data capture and processing description can be found in the Supplementary material.

High-throughput phenotyping data consisting of five color bands (red, green, blue, NIR, and RedEdge) was available for three field seasons in the two locations throughout the vegetation period, however, with varying temporal resolution: from 4 to 22 missions (Table 1).

**Table 1:** HTP mission overview: number of data capture sessions for each year, camera, and location

Year	Camera and location			
	Micasense RedEdge M		Phantom 4 Multispectral	
	Vollebekk	Staur	Vollebekk	Staur
2019	7	4	-	-
2020	12	-	-	-
2021	8	-	22	-

**Fig. 1:**
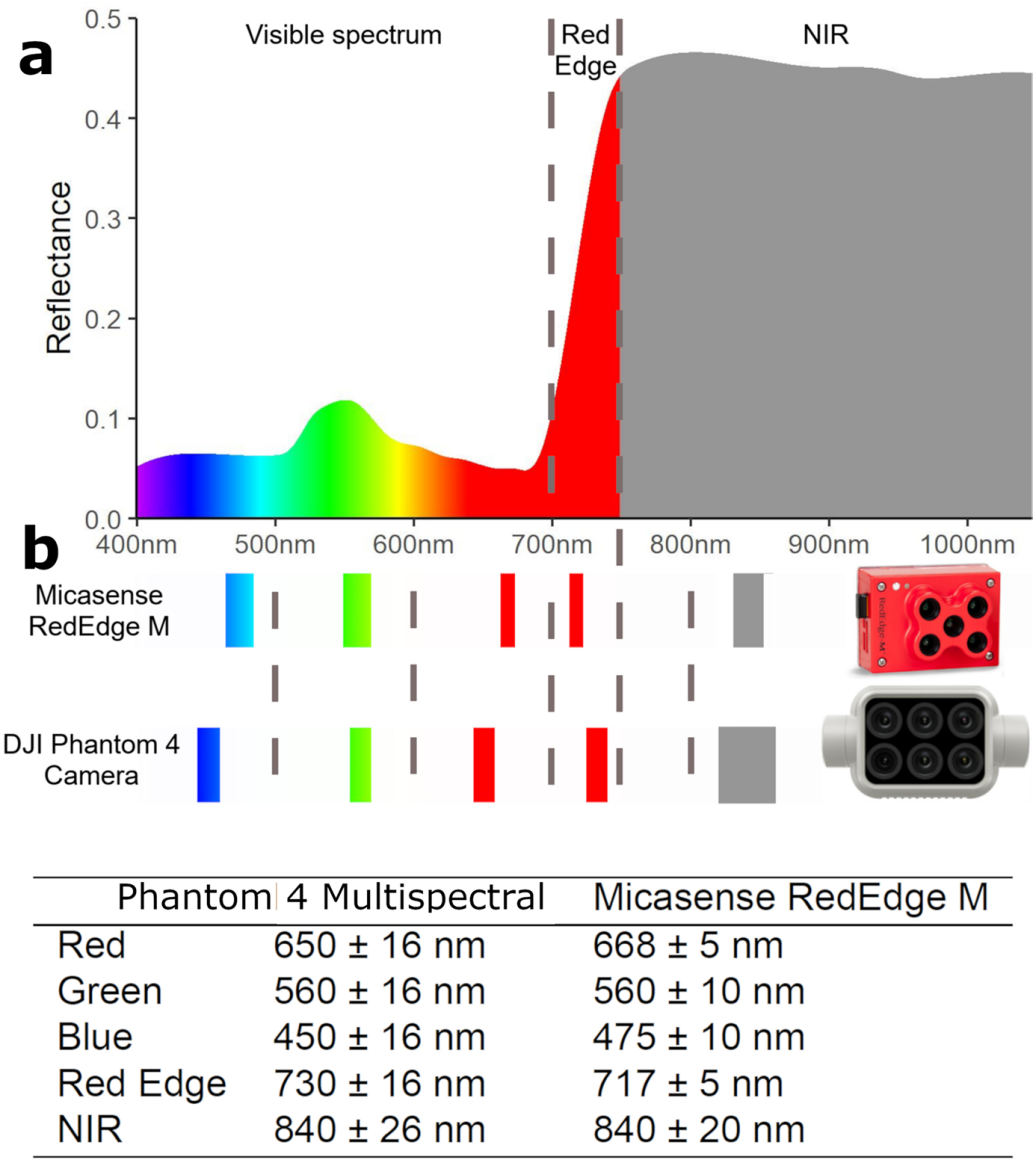
**a** Typical plant canopy reflectance spectrum with graphical interpretation of light spectrum wavelengths. **b** Visual interpretation and numeric values of central bands and bandwidths for the two tested cameras: Micasense RedEdge M, and Phantom 4 Multispectral

The two cameras are fundamentally different regarding resolution and bandwidths/central bands (Fig. 1), so they were analyzed separately. Only raw canopy reflectance values (red, green, blue, NIR, and RedEdge) were used for every part of the analysis, without calculating multispectral indices.

Raw reflectance values in each of the environments and cameras are shown in Fig. S1. Correlation coefficients of raw reflectance values with GY in each of the environments are displayed on Fig. S2.

### Analyzed models

Several models described below were developed and tested using R package “lme4GS” (Caamal-Pat et al. 2021) in R version 4.2.1.

#### G-single-environment genomic prediction

To benchmark single-environment analysis, genomic prediction using G (genomic kinship) matrix (G-BLUP, according to VanRaden, 2008) was performed in single-environment scenario (“Model performance assessment”). G was calculated according to Eq. (5):

5 $$G=\frac{W{W}{\prime}}{n}$$

where *n* is the number of genotypes; *G* is the square genomic relationship matrix with *n* rows and *n* columns corresponding to the genotypes; $W$ is a scaled (mean = 0, standard deviation = 1) matrix of SNP marker data with *n* rows and *m* columns (which equals number of quality-checked markers, coded as 0s and 2s); and ${W}{\prime}$ is its transpose.

For every environment (year/location combination), a random effect model was fitted using G as the definition of variance/covariance structure among the genotypes according to Model 1:

Model 1 $$y=\mu 1+g+e$$

where $y$ is the vector of BLUEs for a trait for *n* genotypes, $\mu$ is the intercept, $1$ is a vector of ones, $g$ is the vector of random genotypes effects distributed as $g\sim N(0,G{\sigma }_{g}^{2})$, and $e$ is the vector of residual effects distributed as $e\sim N(0,{\sigma }^{2})$.

Model 1 was trained and tested on environment (field trial), location, and global BLUEs.

#### G-multi-environment genomic prediction

To benchmark multi-environment prediction using the G matrix, Model 1 was used in the multi-environment scenario (“Model performance assessment”), using only environment (trial) BLUEs.

#### G + E-multi-environment genomic prediction with environment covariance (E) matrix

To benchmark multi-environment predictions using the G matrix coupled with the environmental (phenotypical) variance/covariance matrix K_E_, genomic prediction supplemented with K_E_ matrix was analyzed in a multi-environment scenario (“Model performance assessment”). For this purpose, only environment (trial) BLUEs were used.

The K_E_ matrix was computed for GY according to Eq. (6):

6 $${K}_{E}=\frac{P{P}{\prime}}{n}$$

where *n* is the number of environments (environment/season combinations), ${K}_{E}$ is the square environmental (phenotypical) variance/covariance matrix for GY of dimensions *n* × *n*, *P* is a scaled rectangular matrix with *n* rows and *m* columns (representing scaled phenotype values for every genotype for every environment in rows), and ${P}{\prime}$ is its transpose.

Using *G* and *E*, Model 2 was fitted:

Model 2 $$y=\mu 1+E+g+e$$

All the terms of Model 2 are equal to Model 1 except for *E*—vector of random environment effects, $E\sim N(0,{K}_{E}{\sigma }_{E}^{2})$.

#### M-single-environment prediction using image-derived M matrix

For every environment, based on BLUE values for every available raw band for each flight date and each genotype (“Statistical analysis of the field trial data”), a multispectral relationship matrix was computed according to Eq. (7) and analogically to G and E matrices and similar to the work of Krause et al. (2019):

7 $${K}_{M}=\frac{C{C}{\prime}}{n}$$

where *n* is the number of genotypes, ${K}_{M}$ is the multispectral variance/covariance matrix of dimensions *n* × *n* in a particular season, *C* is a scaled rectangular matrix with *n* rows and number of columns corresponding to genotypic BLUE reflectance values for each multispectral band at every flight within the season, and ${C}{\prime}$ is its transpose.

As the reflectance values are assumably environment-specific, the ${K}_{M}$ matrix was computed for each environment (year/location combination) separately, with no attempt to calculate a cross-environment ${K}_{M}$ matrix.

Using the derived ${K}_{M}$ matrix, an analogical analysis to single-environment genomic prediction was conducted by replacing *G* with ${K}_{M}$ matrix in a single-environment scenario (“Model performance assessment”) and fitting Model 3:

Model 3 $$y=\mu 1+{g}_{*}+e$$

where ${g}_{*}$ is the vector of random genotype effects distributed as $g\sim N\left(0,{K}_{M}{\sigma }_{{g}_{*}}^{2}\right).$ Each ${K}_{M}$ matrix (developed based on different environment data) was trained and tested on environment, location, and global BLUEs.

#### Multi-environment prediction using M matrix

To assess whether ${K}_{M}$ matrix derived based on data from a single season possesses prediction ability in other environments (if it is environment-specific), an analogical analysis was carried out in a multi-environment scenario (“Model performance assessment”) by replacing G with ${K}_{M}$ in Model 1. Each environment’s ${K}_{M}$ matrix was tested for its multi-environment prediction ability using only environment (trial) BLUEs. There was no attempt to calculate a cross-environment ${K}_{M}$ because of the assumed environment-specificity of multispectral data.

#### Multi-environment prediction using M and E matrices

An analogical model to Model 2 was tested in a multi-environment scenario (“Model performance assessment”) by replacing *G* with the ${K}_{M}$ matrix. For this purpose, only environment (trial) BLUEs were used.

#### G + M-single-season genomic prediction supplemented with M matrix

To assess the prospect of supplementing genomic prediction models with multispectral data, Model 4 combining both *G* and ${K}_{M}$ matrices was fitted for a single-environment scenario (“Model performance assessment”):

Model 4 $$y=\mu 1+g+{g}_{*}+e$$

with terms identical as in Models 1 and 3. For the purpose, BLUEs over environments were used.

#### G + M-multi-environment genomic prediction supplemented with M matrix

To evaluate the combined prediction ability of the *G* and ${K}_{M}$ matrices, Model 4 was tested in the multi-environment scenario (“Model performance assessment”) using only environment (trial) BLUEs.

#### G + M + E-multi-environment genomic prediction supplemented with M and E matrices

To further evaluate the combined prediction ability of *G* and ${K}_{M}$ matrices in multi-environmental scenario (“Model performance assessment”), Model 5 was developed using *G*, *M*, and *E* matrices simultaneously:

Model 5 $$y=\mu 1+E+g+{g}_{*}+e$$

with terms identical as in the previous models. For this purpose, only environment (trial) BLUEs were used.

### Model performance assessment

The models’ performance was analyzed under two scenarios (described below): single (G, M, and G + M) and multi-environment (G, M, G + E, G + M, M + E, and G + M + E). The assessment was performed using the following metrics:

rTRN—prediction ability in the training set (in the dataset used to develop the model), defined as the Pearson correlation coefficient between predicted and observed values.

rTST—prediction ability in the testing set (the dataset not seen previously by the model), defined as the Pearson correlation coefficient between predicted and observed values.

rmseTRN—root mean squared error in the training set, defined as

$${{\text{rmse}}}_{{\text{TRN}}}=\sqrt{\frac{\sum_{n=1}^{N}{({{\text{obs}}}_{{\text{TRN}}}-{{\text{pred}}}_{{\text{TRN}}})}^{2}}{N}}$$

where obs_TRN_ are observed (ground truth) phenotypes, pred_TRN_ are predicted phenotypes (output from the models), and *N* is the number of records (genotypes) in the training set.

rmseTST is root mean squared error in the test set (previously unseen data), defined as

$${{\text{rmse}}}_{{\text{TST}}}=\sqrt{\frac{\sum_{n=1}^{N}{({{\text{obs}}}_{{\text{TST}}}-{{\text{pred}}}_{{\text{TST}}})}^{2}}{N}}$$

where obs_TST_ are observed (ground truth) phenotypes, pred_TST_ are predicted phenotypes (output from the models), and *N* is the number of records (genotypes) in the testing set.

The models were tested using cross-validation with 200 iterations in two scenarios:

Single environment: the training set consisted of 80% of genotypes available in the respective environment/mean (20% as testing set). Genotypes were randomly assigned to training/test sets at every iteration.

Multi-environment: the testing set consisted of 20% of all the available genotypes in two environments not used for training the model. The training set comprised 80% of all the available genotypes in the remaining environments (9). Therefore, the testing set was double-blind: comprised of both environments and genotypes not used for model training. Both genotypes and environments were randomly assigned to training/testing sets at every iteration.

### The importance of camera bands for GY prediction

Model 3 was tested in a single-environment scenario with M matrices constructed based on all flight times with only a single camera band at a time (red, green, blue, RedEdge, and NIR) to verify the importance of camera bands for GY prediction using the M matrix.

### The importance of timing of data capture

Model 3 was tested in a single-environment scenario with M matrices constructed on all camera bands but with only one date at a time to verify the effect of time of data capture on GY prediction ability.

### Minimal setup for GY prediction

Based on the results mentioned in the previous paragraphs, a concept of minimal setup for GY prediction was formed: a single flight mission taken during July (grain filling stage). This concept was developed for multispectral cameras (with five bands) and a simple RGB camera (3 bands, red, green, and blue). The RGB camera was “simulated” using only three bands (out of the five available bands) for constructing M matrices.

Model 3 was tested in the single-environment scenario, constructing M matrices based on a random flight date in July in each environment with five (multispectral camera) or three (RGB camera) bands.

## Results

### Phenotypic data evaluation—GY

**Fig. 2:**
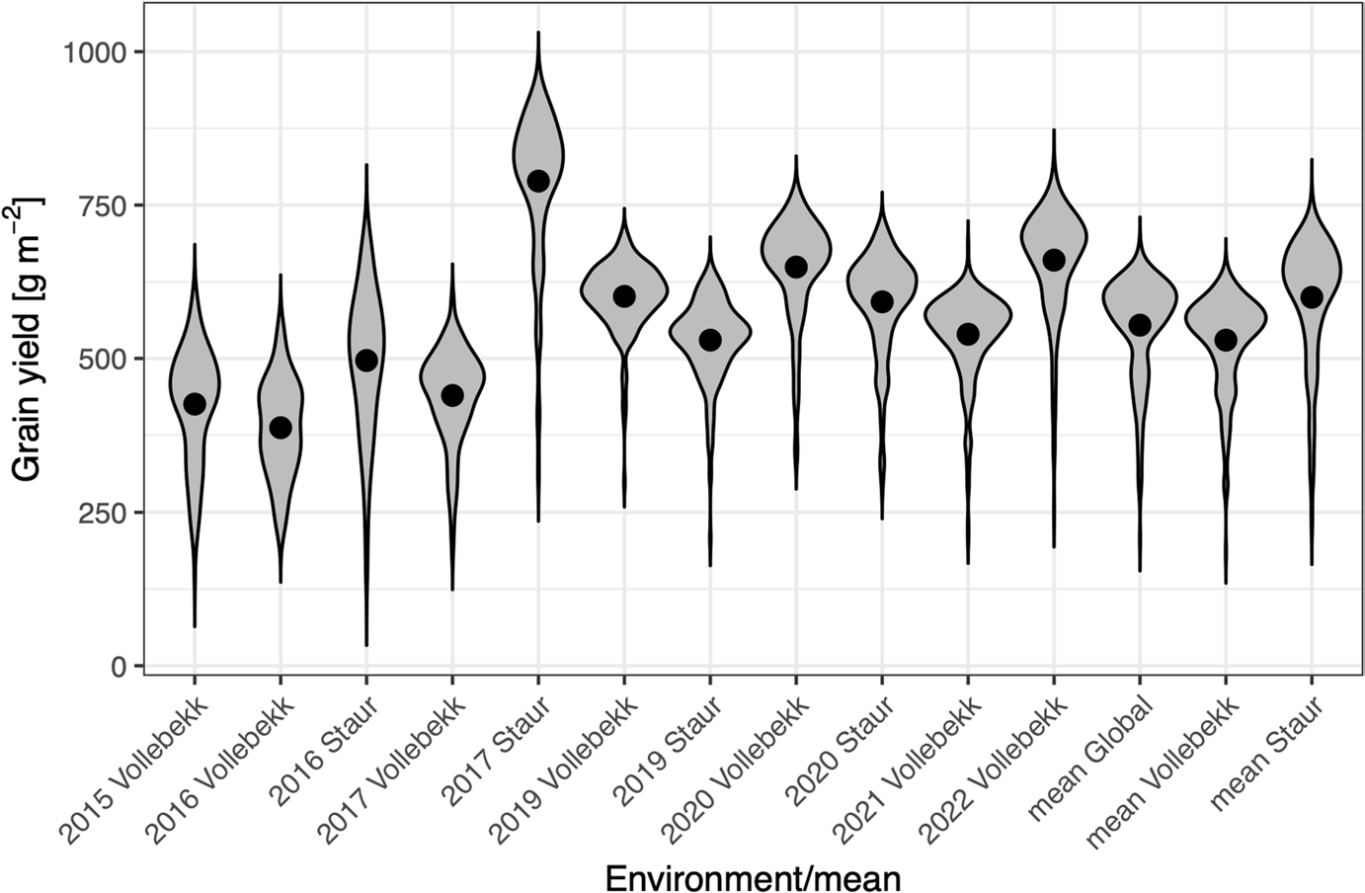
Violin plot of distributions of GY genotypical means in each of the studied environments and means: global means (across all studied environments) and location specific means (across all environments within one location: Staur or Vollebekk). Black dots indicate mean values

Mean genotypic GY values across all environments (year and location combinations) are similar (approximately 520 g m^−2^), except for a field experiment in Staur in 2017 when the average GY value reached 789 g m^−2^. The global mean is influenced mainly by trials conducted in Vollebekk and resembles the distribution of the Vollebekk environment mean. The environment mean in Staur is higher than the Vollebekk means by 70 g m^−2^. In all environments and means, a long left tail can be observed in the distributions (Fig. 2).

Across the field trials (environments), broad-sense heritability for GY ranged from 0.63 (2017 Vollebekk) to 0.92 (Vollebekk 2015) and the number of tested genotypes varied between 98 and 296 (Table 2).

**Table 2:** Broad sense heritability (*H*^2^) of GY in each environment and number of genotypes (*n* genotypes) tested in each environment (field trial)

Environment	*n* genotypes	*H*^2^
2015 Vollebekk	157	0.92
2016 Staur	100	0.71
2016 Vollebekk	98	0.73
2017 Staur	240	0.83
2017 Vollebekk	240	0.63
2019 Staur	220	0.83
2019 Vollebekk	220	0.81
2020 Staur	288	0.68
2020 Vollebekk	288	0.73
2021 Vollebekk	293	0.84
2022 Vollebekk	296	0.90

**Fig. 3:**
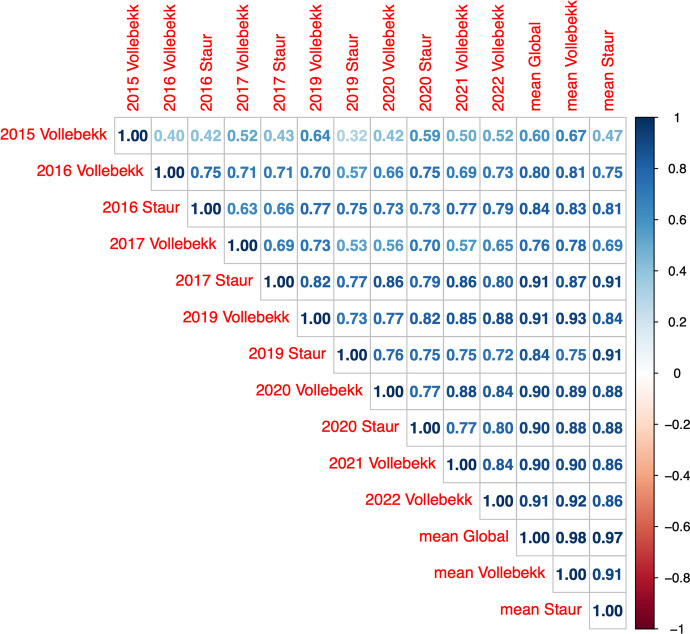
Genotypic Pearson correlations for GY values among field trials (environments), their means, and the global mean

Field trials (environments) and means were, on average, highly correlated (*r* = 0.77). The field trial from Vollebekk in 2015 is the most different from the remaining trials and means, with *r* ranging from 0.32 (with Staur 2019) to 0.64 (with Vollebekk 2019) and 0.67 with the Vollebekk environmental mean. The location means resemble more recent trials (2019 onwards), which can also be observed for the global mean (Fig. 3).

### High-throughput phenotyping data evaluation

Raw reflectance values for each band over field seasons in each environment are shown on Fig. S1.

**Fig. 4:**
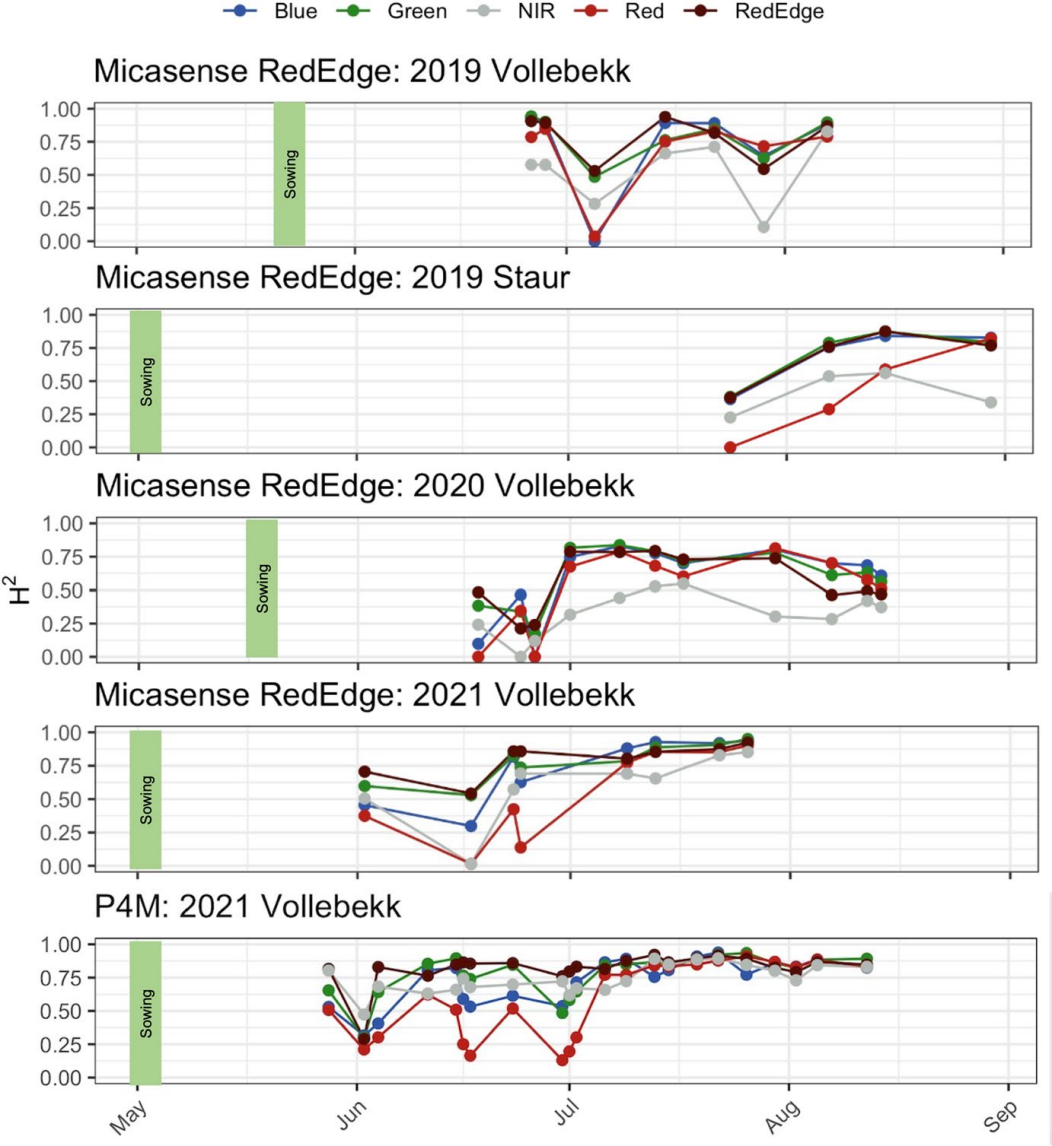
Broad-sense heritability estimates for each band, in each environment, and for each flight and camera. The *X* axis, data capture date (flight date); *Y* axis, broad sense heritability. Line colors correspond to the bands they represent (RGB); gray, NIR; dark red, RedEdge

Broad-sense heritability of each band changed during the season with no apparent consistent trend; however, heritability values tended to be more stable later in the growing season (from July onwards). NIR and red were the least heritable bands, while RedEdge, green, and blue had higher heritability values. It was not uncommon to observe that during the same mission, different bands had very different heritabilities (Fig. 4).

### Evaluation of single environment prediction using G matrix

GY prediction using the G matrix in single-environment scenarios (model trained and validated on a single season) consistently showed high prediction ability in the training set (on average 0.99). In contrast, accuracies in testing sets ranged from 0.59 to 0.81 in individual field trials, averaging to 0.75. In both location and the global BLUEs, where the genetic signal is stronger, testing accuracies (rTST) were higher than in the individual trials. Root mean squared error (rmse) in the testing set was approximately four times higher than in the training set (53.0 and 13.9 for testing and training sets, respectively, Table 3).

**Table 3:** Comparison of GY prediction ability and root mean squared error for G matrix in a single season scenario (models built and verified on a single environment) using cross-validation with 200 iterations. rTRN prediction ability in the training set, rTST prediction ability in the testing set, rmseTRN root mean squared error in the training set, rmseTST root mean squared error in the testing set

Environment/mean	G matrix			
	rTRN	rTST	rmseTRN	rmseTST
2015 Vollebekk	0.99	0.69	15.7	64.9
2016 Staur	0.99	0.75	14.9	73.6
2016 Vollebekk	0.98	0.72	14.7	52.0
2017 Staur	0.99	0.81	19.7	69.7
2017 Vollebekk	0.98	0.70	16.2	52.4
2019 Staur	0.95	0.59	23.6	51.9
2019 Vollebekk	0.98	0.63	11.1	40.7
2020 Staur	0.99	0.77	12.6	51.9
2020 Vollebekk	0.98	0.75	17.9	53.5
2021 Vollebekk	0.99	0.75	9.9	46.0
2022 Vollebekk	0.99	0.79	14.4	51.5
Mean global	1.00	0.86	6.3	40.2
Mean Staur	0.99	0.83	14.5	54.4
Mean Vollebekk	1.00	0.85	3.6	39.1
Avg	0.99	0.75	13.9	53.0

### Evaluation of single environment prediction using M matrix

**Fig. 5:**
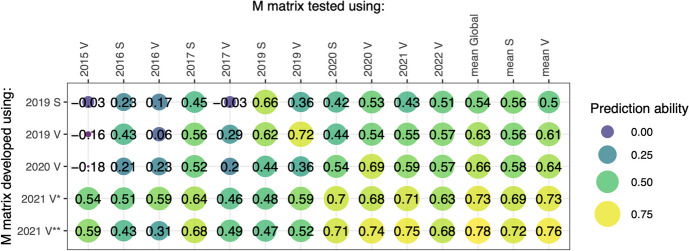
Prediction abilities achieved in single-environment scenarios by using M matrix constructed on multispectral data from each environment with multispectral data available. The *Y* axis indicates from where the environment multispectral data originated, while the *X* axis indicates the environment on which the model was trained and tested. Numbers and colors indicate the prediction ability in the testing set defined as Pearson correlation between predicted and actual values. *Data gathered using Micasense RedEdge camera; **data gathered using Phantom 4 Multispectral camera. Abbreviated location names: S Staur, V Vollebekk

M matrices showed the highest prediction ability on the environment they originated from; however, they often retained prediction ability when tested on other environments, especially those highly correlated with their environment of origin. M matrices developed in seasons 2019 and 2020 showed poor prediction ability in 2015 Vollebekk, 2016 Vollebekk, and 2017 Vollebekk due to low correlations with those environments. M matrices developed with data from 2021 Vollebekk (using both cameras) showed decent prediction abilities across all the tested environments, even in environments not strongly correlated with the M matrix’s origin (Fig. 3). Prediction abilities were high (> 0.5) for the global and location means for all the M matrices (Fig. 5).

### Evaluation of G, M, and G + M models in single-environment scenarios

Genomic prediction accuracies in testing sets ranged from 0.59 to 0.75, averaging 0.68 in the chosen environments, while training set accuracies reached nearly perfect (0.98). Predictions using the M matrix in the single-environment scenario showed, on average, lower training set prediction abilities than predictions using the G matrix (0.79 and 0.98, respectively); however, testing set prediction abilities were higher than those of the G matrix (0.71 and 0.68, respectively). By comparing the difference between training and testing set prediction abilities, the M matrix model was less prone to overfitting than the model using the G matrix (difference of 0.08 and 0.30 for M and G matrices, respectively) (Table 4).

**Table 4:** Comparison among prediction abilities of M matrices originating from different seasons, prediction using only the G matrix (genomic prediction), and a combined model utilizing both G and M matrices in the same model in the single-environment scenario. Models were developed and tested on single environment (trial) BLUEs. Abbreviated location names: *S* Staur, *V* Vollebekk, *rTRN* prediction ability in the training set, *rTST* prediction ability in the testing set, *rmseTRN* root mean squared error in the training set, *rmseTST* root mean squared error in the testing set. *M matrix developed using Micasense RedEdge M camera data; **M matrix developed using Phantom 4 Multispectral camera data

Model	Metric	Season/M matrix origin (if M present)					Avg
		2019 S	2019 V	2020 V	2021 V*	2021 V**	
G	rTRN	0.95	0.98	0.98	0.99		0.98
	rTST	0.59	0.63	0.75	0.75		0.68
	rmseTRN	23.61	11.06	17.87	9.88		15.61
	rmseTST	51.88	40.72	53.51	45.95		48.02
M	rTRN	0.74	0.81	0.75	0.81	0.84	0.79
	rTST	0.66	0.73	0.69	0.71	0.75	0.71
	rmseTRN	43.20	31.37	53.62	40.24	38.07	41.30
	rmseTST	48.84	37.03	58.13	49.11	45.90	47.80
G + M	rTRN	0.96	0.98	0.99	1.00	1.00	0.99
	rTST	0.74	0.79	0.83	0.80	0.81	0.79
	rmseTRN	19.51	11.02	11.23	6.87	6.19	10.96
	rmseTST	42.54	32.33	45.76	41.00	40.96	40.52

Supplementing genomic prediction (G matrix) with the M matrix in a single-environment scenario yielded similar prediction ability (0.71 for M versus 0.68 for G). The G + M model exhibited traits of both individual matrix models and performed better than either G or M alone: very high training prediction ability, high testing prediction ability, low training set error, and low testing set error (Table 4).

### Evaluation of G, M, G + M, G + E, and G + M + E models in multi-environment scenarios

GY prediction using the G matrix alone in multi-environmental scenarios achieved accuracies of 0.57 and 0.49 in training and testing sets, respectively. Prediction ability in testing sets using M matrices originating from different environments ranged from 0.27 to 0.44, averaging 0.36. Replacing G with the M matrix resulted in a considerable reduction of prediction ability (difference in testing prediction ability of 0.13) and a slightly larger degree of overfitting of the model (difference of prediction ability in training–testing sets of 0.12) (Table 5).

**Table 5:** Comparison of prediction ability of different models including combinations of G, E, and M matrices in the multi-environmental scenarios (two environments as testing sets, drawn randomly at every iteration). M matrices were developed based on data originating from different environments, and each M matrix has been tested individually on randomly selected test environments over 200 iterations. Abbreviated location names: *S* Staur, *V* Vollebekk, *rTRN* prediction ability in the training set, *rTST* prediction ability in the testing set, *rmseTRN* root mean squared error in the training set, *rmseTST* root mean squared error in the testing set. *M matrix developed using Micasense RedEdge M camera data; ** M matrix developed using Phantom 4 Multispectral camera data

Model	Metric	If M present, M matrix developed on data from					Avg
		2019 S	2019 V	2020 V	2021 V*	2021 V**	
G	rTRN	0.57					-
	rTST	0.49					-
	rmseTRN	111.1					-
	rmseTST	117.0					-
M	rTRN	0.43	0.40	0.54	0.52	0.52	0.48
	rTST	0.27	0.32	0.36	0.42	0.44	0.36
	rmseTRN	110.6	111.6	112.2	113.4	113.5	112.3
	rmseTST	117.0	115.0	124.2	121.7	119.7	119.5
G + E	rTRN	0.95					-
	rTST	0.83					-
	rmseTRN	40.59					-
	rmseTST	58.16					-
G + M	rTRN	0.46	0.46	0.56	0.57	0.57	0.52
	rTST	0.44	0.46	0.62	0.57	0.63	0.56
	rmseTRN	107.0	107.0	110.0	108.0	109.0	108.0
	rmseTST	121.0	121.0	117.0	124.0	121.0	120.8
M + E	rTRN	0.90	0.92	0.90	0.91	0.93	0.91
	rTST	0.70	0.78	0.73	0.77	0.79	0.75
	rmseTRN	50.8	45.9	56.7	53.7	49.0	51.3
	rmseTST	62.3	57.4	68.6	63.4	60.3	62.4
G + E + M	rTRN	0.95	0.95	0.95	0.95	0.95	0.95
	rTST	0.76	0.79	0.84	0.84	0.85	0.82
	rmseTRN	36.9	37.2	40.1	40.2	40.5	39.0
	rmseTST	54.9	50.9	56.2	54.8	53.0	53.9

Supplementing genomic prediction with a phenotypically derived E matrix drastically increased the prediction ability in training and testing sets (0.95 and 0.83, respectively) and reduced the errors. The G + E model achieved the highest prediction ability among all the tested models (Table 5).

Aiding genomic prediction with M matrices also increased the prediction ability, albeit smaller than adding the E matrix (testing sets prediction ability difference of 0.27 between G + E and G + M models). Prediction based on M matrices coupled with the E matrix achieved a prediction ability comparable with genomic prediction aided by the E matrix (testing set accuracies of 0.75 and 0.83 for M + E and G + E models, respectively). The M + E model was similar to the G + E model in its degree of overfitting (difference between training and testing set accuracies of 0.12 and 0.16, respectively) (Table 5).

The most complex model, utilizing G, E, and M matrices, achieved prediction abilities almost identical to the G + E model (testing set accuracies of 0.82 and 0.83, respectively); however, adding multispectral information resulted in minor errors both in training and testing sets, as compared to the G + E model. M matrix originating from the 2021 Vollebekk environment (with the highest temporal density) paired with G and E matrices showed the highest prediction ability in the prediction of GY (testing set prediction ability 0.85) (Table 5).

### Which camera bands are the most informative for GY prediction using M matrix?

In single-environment scenario, GY prediction using a constructed M matrix based on only one band reduced prediction ability by 35% compared to the entire M matrix (average testing set prediction ability for the individual bands of 0.46 compared to 0.71 for the entire M matrix, Table 4 and 5). On average, bands exhibited the following ranking (descending prediction in the test set prediction ability): RedEdge, red ex aequo green and blue, and NIR; however, these differed slightly among the environments. The bands with the highest prediction abilities were RedEdge and the three “basic” bands (red, green, and blue). Contrastingly, the least informative band was consistently NIR (except for Vollebekk 2019, where it ranked 4), with high variability in the testing set prediction ability reaching as low as − 0.22 in the Staur 2019 environment. The remaining bands were consistent in their prediction abilities (Table 6).

**Table 6:** Comparison of prediction abilities of constructed M matrices based on a single band captured during a single season in a single-environment scenario. Abbreviated location names: *S* Staur, *V* Vollebekk, *rTRN* prediction ability in the training set, *rTST* prediction ability in the testing set, *rmseTRN* root mean squared error in the training set, *rmseTST* root mean squared error in the testing set. *M matrix developed using Micasense RedEdge M camera data; ** M matrix developed using Phantom 4 Multispectral camera data

Band	Metric	Environment					Avg
		2019 S	2019 V	2020 V	2021 V*	2021 V**	
Red	rTRN	0.59	0.58	0.64	0.59	0.73	0.63
	rTST	0.55	0.47	0.60	0.44	0.63	0.54
	rmseTRN	52.29	44.2	62.6	55.95	47.2	52.4
	rmseTST	52.7	46.5	66.0	70.2	54.8	58.0
Green	rTRN	0.59	0.62	0.59	0.59	0.71	0.62
	rTST	0.55	0.56	0.53	0.54	0.65	0.53
	rmseTRN	52.4	42.4	65.6	56.0	48.6	53.0
	rmseTST	53.1	44.8	68.1	59.0	52.2	55.4
Blue	rTRN	0.45	0.38	0.60	0.57	0.69	0.54
	rTST	0.40	0.25	0.53	0.47	0.65	0.46
	rmseTRN	57.4	50.2	65.2	56.5	50.1	55.9
	rmseTST	59.6	51.9	67.7	63.6	52.9	59.1
RedEdge	rTRN	0.63	0.63	0.58	0.60	0.69	0.63
	rTST	0.60	0.58	0.53	0.56	0.61	0.58
	rmseTRN	50.2	42.0	66.3	54.9	49.7	52.6
	rmseTST	51.4	44.0	68.6	59.4	55.8	55.8
NIR	rTRN	0.82	0.45	0.42	0.44	0.66	0.56
	rTST	-0.22	0.35	0.21	0.13	0.55	0.21
	rmseTRN	30.7	48.4	73.0	58.0	51.8	52.4
	rmseTST	64.6	50.6	81.2	68.7	58.8	64.8

Bearing similarity to the single-environment scenarios (Table 6), M matrices developed on single bands had poor and reduced prediction ability in multi-environment scenarios by 41% (average testing set prediction ability of 0.21) compared to the entire M matrices (Table 5 and 7). The average ranking of bands also bared similarity to the single-environment scenarios: RedEdge *ex aequo* red and green, blue, and NIR.

**Table 7:** Comparison of prediction ability of constructed M matrices based on a single camera band in multi-environment scenarios (two environments as testing set, drawn randomly at every iteration). The development of M matrices was based on data originating from different environments, and each M matrix has been tested individually on randomly selected test environments over 200 iterations. Abbreviated location names: *S* Staur, *V* Vollebekk, *rTRN* prediction ability in the training set, *rTST* prediction ability in the testing set, *rmseTRN* root mean squared error in the training set, *rmseTST* root mean squared error in the testing set. *M matrix developed using Micasense RedEdge M camera data; ** M matrix developed using Phantom 4 Multispectral camera data

Band	Metric	M matrix developed on data from:					Avg
		2019 S	2019 V	2020 V	2021 V*	2021 V**	
Red	rTRN	0.46	0.44	0.56	0.56	0.55	0.51
	rTST	0.21	0.24	0.24	0.25	0.35	0.26
	rmseTRN	109.6	110.3	111.3	111.4	112.1	111.0
	rmseTST	119.7	118.1	128.5	134.3	124.7	125.0
Green	rTRN	0.46	0.45	0.56	0.57	0.55	0.52
	rTST	0.22	0.23	0.23	0.25	0.35	0.26
	rmseTRN	109.3	110.0	111.4	110.9	111.7	111.0
	rmseTST	119.2	118.6	128.8	130.4	124.7	124.0
Blue	rTRN	0.48	0.48	0.57	0.58	0.57	0.53
	rTST	0.10	0.12	0.18	0.12	0.26	0.16
	rmseTRN	109.1	109.3	111.1	110.6	111.1	110.0
	rmseTST	121.0	121.4	131.7	133.1	129.2	127.0
RedEdge	rTRN	0.44	0.45	0.56	0.56	0.56	0.52
	rTST	0.24	0.25	0.24	0.26	0.33	0.26
	rmseTRN	110.1	110.0	111.27	111.15	111.5	111.0
	rmseTST	118.1	117.7	128.8	129.3	125.9	124.0
NIR	rTRN	0.48	0.47	0.57	0.58	0.56	0.53
	rTST	0.00	0.18	0.08	0.10	0.31	0.13
	rmseTRN	109.2	109.5	110.9	110.5	111.5	110.0
	rmseTST	122.8	120.3	132.7	133.3	127.2	127.0

### Effect of multispectral data capture on GY prediction ability

**Fig. 6:**
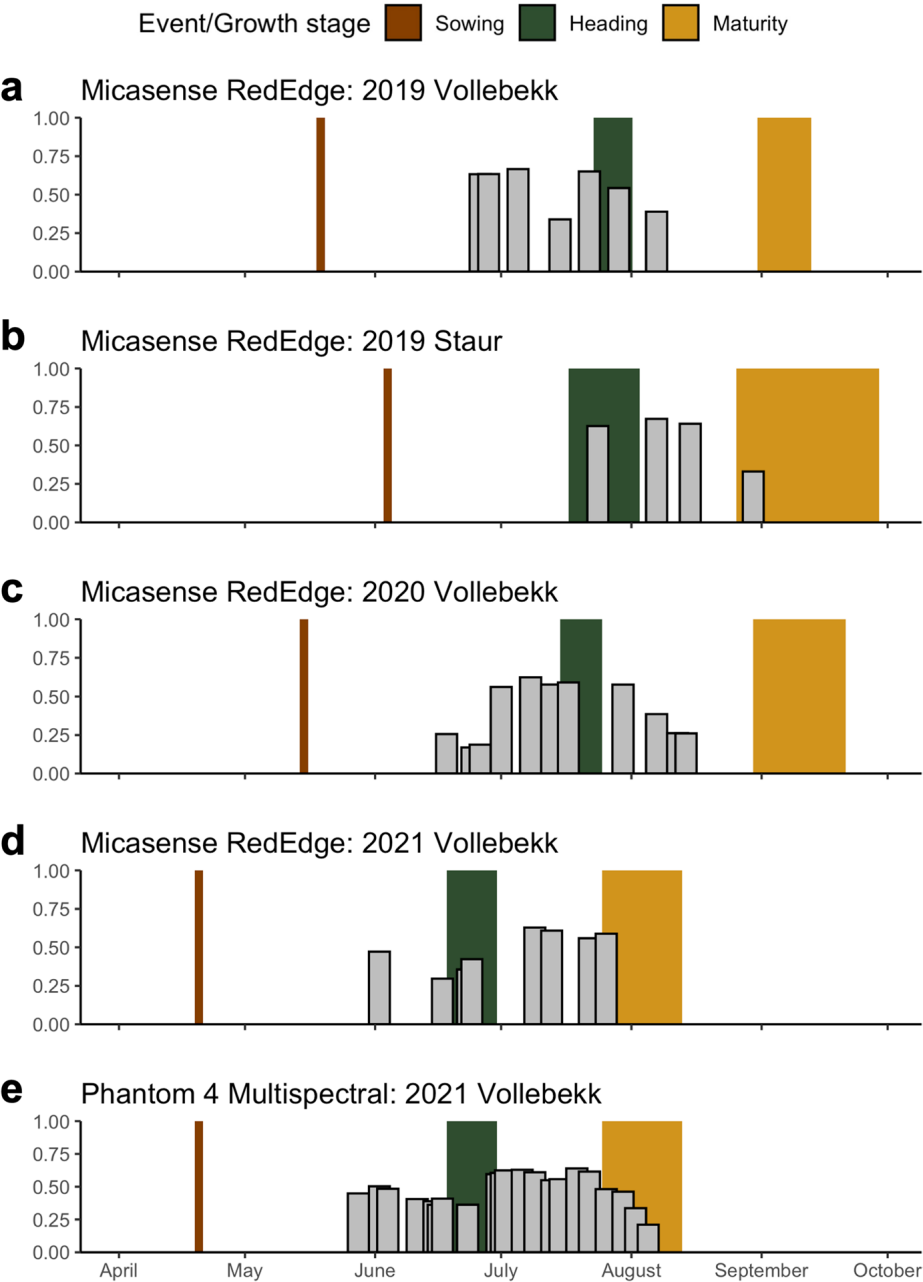
Prediction ability of M matrix developed on all bands from a single date in single-environment scenario, in environments 2019 Vollebekk (**a**), 2019 Staur (**b**), 2020 Vollebekk (**c**), and 2021 Vollebekk (**d**, **e**). Phenotyping data was gathered using Micasense RedEdge (**a**–**d**) or Phantom 4 Multispectral (**e**) camera. Colored regions in the plots indicate approximate growing stages of the panel in each environment. *X* axis, date of mission; *Y* axis, prediction ability

GY prediction in single-environment scenarios was possible, with accuracies ranging from 0.17 to 0.68. Based on all the environments, data capture sessions late in the growing season (when plants approach physiological maturity) tended to be less informative (Fig. 6). At the same time, the prediction ability dropped further as maturing progressed. It is difficult to conclude the informativeness of early season flights due to the scarcity of available records; however, based on Vollebekk 2020 and 2021 environments, early season flights are more informative than flights taken later, until approximately the end of June. Data capture sessions carried out in July showed the highest prediction ability in all the seasons with stable accuracies (Fig. 6). Based on the 2021 Vollebekk environment, no meaningful differences in prediction ability could be observed between the two used cameras (Fig. 6).

### Minimal GY prediction setup in single-environment scenarios using the M matrix

The prediction ability ranged from 0.51 to 0.58 and 0.55 to 0.62 for RGB and MS cameras, respectively—using MS instead of RGB cameras increased prediction ability only slightly (average difference of 0.04). Prediction performed using both cameras showed identical degrees of overfitting (prediction ability difference between training and testing sets of 0.03) (Table 8).

**Table 8:** Comparison of GY prediction ability in single-environment scenarios using a minimal setup (single data capture during July) with M matrices developed based on RGB and multispectral data (cameras). Abbreviated location names: S Staur, V Vollebekk, rTRN prediction ability in the training set, rTST prediction ability in the testing set, rmseTRN root mean squared error in the training set, rmseTST root mean squared error in the testing set. *M matrix developed using Micasense RedEdge M camera data; ** M matrix developed using Phantom 4 Multispectral camera data

Setup	Metric	M matrix developed on data from:					Avg
		2019 S	2019 V	2020 V	2021 V*	2021 V**	
RGB camera	rTRN	0.58	0.55	0.60	0.57	0.58	0.58
	rTST	0.56	0.51	0.58	0.54	0.55	0.55
	rmseTRN	53.0	44.3	65.0	56.4	56.1	55.0
	rmseTST	52.4	45.2	66.0	57.2	27.3	55.6
MS camera	rTRN	0.63	0.59	0.62	0.63	0.61	0.62
	rTST	0.62	0.55	0.59	0.60	0.58	0.59
	rmseTRN	50.4	42.4	63.7	53.4	54.3	52.9
	rmseTST	50.5	44.3	65.2	54.7	56.0	54.1

## Discussion

Using relationships among objects or individuals has been present in plant breeding for over 100 years and has been the foundation for quantitative genetics. Those relationships can be derived based on various properties of the individuals, such as their pedigrees (A matrix) or dense genotyping data (Meuwissen, Hayes, and Goddard, 2001), and are widely used in breeding programs worldwide for both simple and complex traits. This study elaborates on utilizing multispectral phenotypes to construct genotypic relationships. The described methodology, bearing similarity to the G or A matrices or work of Krause et al. (2019), focuses not on individual numerical multispectral phenotype values and their possible abstract relationship with the complex trait of interest but rather on the similarities between genotypes.

Having standard GS in a single-environment scenario as a benchmark, M matrix-based prediction performed similar. GS tended to reach almost perfect prediction ability in the training set, with a considerable drop in prediction ability when tested on new lines. With an average GY heritability of 0.6 in this study, we can see that GS attempts to predict experimental error in individual trials. The prediction ability difference between training and testing sets for the M matrix was four times smaller than for GS, indicating a much lower level of overfitting, probably due to being “closer” to the actual phenotypes. The prediction accuracies using M matrices in a single-environment scenario are comparable to those using H matrices (Krause et al. 2019). H matrix is analogical to the M matrix but developed on hyperspectral data with 62 bands, covering a spectrum between the 380- and 850-nm region. It indicates that introducing more narrower bands is less valuable than using fewer but broader bands available on commercial “low-cost” cameras such as those used in the study. The prediction accuracies achieved by the M matrices are also similar to other studies using linear and non-linear modeling approaches, including OLS (ordinary least squares), Bayesian methods, and PLS (partial least squares), as well as functional regression (Aguate et al. 2017; Montesinos-López et al. 2017) or machine/deep learning methods (Shafiee et al. 2021).

The prediction capabilities of M matrices, developed in various environments, are expected to be lower when assessed using multi-environment means, as they are closely tied to their respective originating environments. Interestingly, when evaluated against multi-environment means, M matrices derived from temporally dense data exhibit a slightly higher prediction ability compared to trial means of their origin. It could be partially because the means resemble the original environment but could also indicate that even the seemingly environment-specific similarity measure has the potential to generalize the genetic part of the phenotype. It is also highlighted by the M matrices originating from different environments, showing prediction power when tested on different environments (with exceptions). The temporally denser the data, the higher the M matrix’s generalization ability. However, it is not easy to consult this hypothesis with available research.

G and M matrices complement each other—the GS model coupled with the M matrix (G + M) in a single-season scenario achieved higher prediction ability than its components alone. The G + M model has the theoretical advantage of using both genetic information and the outcome of this information in a particular environment, capturing more of the crucial G × E interactions. However, the performance gain of adding M to GS was relatively small and came with valuable error reduction in the testing set. Considering the relatively low expense of acquiring multispectral information and its standalone prediction capacity, it can be a viable addition to the practical applications of GS protocols as also shown in other works (Zhu et al. 2022, Robert et al. 2022b).

In multi-environment scenarios, an M matrix–based prediction was inferior to GS, a logical consequence of the inherent environment specificity of the M matrices, as opposed to the “general” genetic nature of the G matrix. However, the prediction ability of M matrices in multi-environment scenarios tended to increase with the number of data capture sessions, which was not the case for single-environment scenarios. It indicates that a temporally denser M matrix can describe the genetic component of GY, reaching prediction ability almost as high as GS, even though this component is not as crucial for the prediction in a single-environment scenario.

GS supplemented with the M matrix shows overall slightly superior prediction ability compared to the GS or M matrix–based prediction alone; however, this appears to depend on the origin of the M matrix and, probably more importantly, the temporal density of data capture sessions (these two are confounded in this work). Despite higher prediction ability, the G + M model shows higher error values, indicating that providing environment-specific information (M matrix) to GS in multi-environment prediction scenarios brings little value without providing further context.

GY prediction in multi-environment scenarios using G or M matrices with environmental context (E matrix) shows high prediction ability, with GS’s superiority in prediction ability and error. It indicates that both layers of information prove informative when used in the environmental context. Although the model combining G, M, and E variates (G + M + E) is not superior to G + E in terms of prediction ability, it minimizes the error, hinting that even only one field season of HTP data capture can aid GS protocols in providing more accurate genetic estimates of GY in multi-environment scenarios.

The camera bands’ relative ranking of prediction ability indicates that heritability is essential. Both the least heritable and the least important band was near-infrared (NIR), despite its established link with plant physiology (multiple reflections of turgid cell structure (Peñuelas & Filella 1998)). Hypothesizing, NIR reflectance could gain importance when water availability severely limits GY output (drought); however, it is impossible to verify this based on our available data. NIR bands tend to be “unstable” and prone to differences in light conditions during data capture, bearing a significant challenge in field based HTP. This problem is partially solved by introducing normalized vegetation indices (VIs, linear combinations of reflectance values in selected spectral regions) such as NDVI, which are more robust under variable lighting conditions.

The most important bands (RedEdge, red, green, and blue) all link to chlorophyll and are more heritable than NIR. RedEdge points to chlorophyll content (Gitelson et al. 1996), and due to its photochemical properties, chlorophyll absorbs red and blue light while reflecting green. Therefore, it is reasonable to hypothesize that chlorophyll properties and content of a genotype govern the usefulness of the M matrix, following findings made by Krause et al. (2019). It may also be that these associations are spurious—the most influential bands are highly heritable, and the M matrix models may therefore work on a “plants that look alike, yield alike” principle without an actual biological component to it.

The most informative data capture time occurs during the grain-filling period, which aligns well with the hypothesis that chlorophyll properties are captured by the M matrix and govern its predictive ability—during grain-filling, higher chlorophyll content means higher assimilation force and photosynthesis rate, resulting in higher GY (Ghimire et al. 2015; Sid’ko et al. 2017). At the same time, inspecting drone imagery during grain filling indicates that the purely visual differences among trial plots are the smallest. Surprisingly, data capture sessions taken later in the growing season yield lower prediction ability. The moment when plants start maturing is easy to determine visually using HTP imagery due to the decay of chlorophyll and water content (change in color). GY is highly correlated with earliness in the NMBU spring wheat panel (Mróz et al. 2023); hence, it should be reasonably possible to predict GY based on differences in genotype earliness. Our findings contradict this hypothesis, as a decay in prediction ability was observed as maturing progressed. These arguments also support the hypothesis of the M matrix using chlorophyll information proxies to predict GY rather than the “plants that look alike, yield alike” principle. Krause et al. (2019) did not observe a similar relationship: all flights taken during the vegetative season yielded comparable prediction ability.

This study used two cameras for HTP data capture: Micasense RedEdge M and Phantom 4 Multispectral camera. They were analyzed back to back for their prediction ability using the M matrix in all models and scenarios. Our results show no evidence to conclude that there are significant differences in prediction ability between the cameras, despite the different technical specifications and numerical reflectance values obtained. This conclusion aligns with the authors’ previous studies, comparing the same two cameras in parallel mission sessions for GY and biomass prediction using machine learning (Shafiee et al. 2023).

Based on our results, the prediction ability gains of using a multispectral camera over a simple RGB camera are incremental, despite multispectral cameras giving access to the informative RedEdge band. Considering the needed hardware, effort, and other resources for GY prediction, a simple RGB camera is more appealing from a purely economic standpoint. It was also exemplified that as little as a single flight mission with a simple RGB camera during the grain-filling period yields enough data to predict GY with prediction ability over 0.5 in a single-environment scenario. It shows the potential of the method and the potential of HTP in large-scale field trial applications.

The usefulness of GS and GY prediction using the M matrix can hardly be compared, as those two methodologies occupy different application niches in plant breeding: the purpose of GS is an early prediction of genotype’s GEBV to enable efficient screening of early-generation progenies in breeding programs and being able to apply speed breeding. Therefore, the most significant advantage of GS is the ability to estimate GEBVs based on a sample of DNA of a single plant earlier. GY prediction using the M matrix does not have this advantage. Genotypes must be put in field trials to collect their multispectral phenotypes, which can occur only in later-generation progenies in reasonably sized field trials. However, prediction using the M matrix scales very well, as adding more plots does not increase the workload linearly (which is the case in GS). Therefore, GY prediction using the M matrix fits well in the later stages of large-scale breeding programs, allowing the breeder to test a more significant number of variety candidates without expanding their technical base.

One disadvantage of the M matrix and machine learning protocols is their inherent connection with their environment of origin. Environment-specific trait estimates are of little use for breeders unless the environment closely resembles their target population of environments. Nevertheless, it was shown that a constructed M matrix based on dense data from a single environment could generalize (to “see” the heritable signal) and perform well when tested on a multi-environment mean. The fact that the M matrix works synergistically with GS makes it an affordable way to improve GS prediction ability and be used as a standalone tool. An added advantage of M matrix-based prediction or its inclusion into GS protocols is its purely statistical and comprehensible nature paired with using already available software without customization.

## Conclusions

Developing genotypic relationships using high-throughput multispectral data (M matrix) gathered using consumer-grade equipment for GY prediction in wheat was elaborated. A back-to-back comparison of the prediction abilities of genomic selection models, including combinations of G, M, and E matrices, was conducted using multi-environment field trial data and mixed models (BLUP) in single and multi-environment scenarios. M matrix possesses standalone prediction ability similar to the G matrix, and genomic selection models can be improved by including both G and M matrices. The importance of camera bands for grain prediction using the M matrix was discussed, showing that bands with the highest heritability are the most important. The importance of data capture was investigated, demonstrating that imagery taken during grain filling yields the best prediction ability. The study also showed that GY prediction is possible using a simple RGB camera with a slight prediction ability loss. The work contributes to expanding use cases for multispectral high-throughput phenotyping data and shows the potential of using this data for improving genomic selection protocols or standalone GY prediction in large-scale field trials.

### Supplementary Information

Below is the link to the electronic supplementary material.

Supplementary file1 (DOCX 3733 KB)

## Data Availability

Data and code available upon a reasonable request to the main author.

## Abbreviations

BLUE: Best linear unbiased estimator
BLUP: Best linear unbiased predictor
G (matrix): Genomic relationship matrix
GBLUP: Genomic best linear unbiased predictor
GEBV: Genomically estimated breeding value
GS: Genomic selection
GY: Grain yield
HTP: High-throughput phenotyping
M (matrix): Multispectrally derived relationship matrix
MAF: Minor allele frequency
MS: Multispectral (in relation to cameras)
NDVI: Normalized differential vegetation index
NIR: Near infrared
PS: Phenomic selection
RGB: Red, green, blue (camera bands)
UAV: Unmanned aerial vehicle, aka drone
UV: Ultra-violet
VI: Vegetation index
