# Supplementary material for: Multispectral-derived genotypic similarities from budget cameras allow grain yield prediction and genomic selection augmentation in single and multi-environment scenarios in spring wheat
Source: Mol Breed. 2024 Jan 15;44(1):5. doi: 10.1007/s11032-024-01449-w (PMC10789716; doi:10.1007/s11032-024-01449-w)
Supplement: Supplementary file 1 — Supplementary file1 (DOCX 3733 KB) [file 11032_2024_1449_MOESM1_ESM.docx]

Supplementary material

**Multispectral-derived genotypic similarities from budget cameras allow grain yield prediction and genomic selection augmentation in single and multi-environment scenarios in spring wheat**

Tomasz Mróz^1^, Sahameh Shafiee^1^, Jose Crossa^23^, Osval A. Montesinos-Lopez^4^, and Morten Lillemo^1^

*^1^Department of Plant Sciences, Norwegian University of Life Sciences, NO-1432 Ås, Norway*

*^2^International Maize and Wheat Improvement Center (CIMMYT), Km 45, Carretera Mexico Veracruz, CP 52640 Texcoco, Edo. de México, Mexico*

*^3^Colegio de Postgraduados, CP 56230 Montecillos, Edo. de México, Mexico*

*^4^Facultad de Telemática, Universidad de Colima, Colima, Mexico*

## Multispectral image acquisition and processing

Two multispectral cameras were used in this study. The RedEdge-M has five imaging sensors, including red, green, blue, red edge, and near InfraRed (NIR) bands (Figure 1). The camera has a 5.4 mm focal length, the sensor size is 4.8 mm x 3.6mm, and the image size is 1280 x 960 pixels. A sunshine sensor can record the illumination information of each image to calibrate the multispectral images. The camera was mounted on a fully programmable DJI Matrix 100 UAV with a maximum payload of 1.25 kg. The flight routes were planned in the Altizure application (Everest Innovation Technology).

The P4M camera has six imaging sensors, five narrow bands, and one combined RGB sensor. The multispectral bands include Red, Green, Blue, red edge, and (NIR). The camera focal length is 5.74 mm, the sensor size is 4.87 mm x 3.96 mm, and the image size is 1600×1300 pixels. This camera is integrated into its UAV platform. Flight routes were planned in the DJI GSPro application (https://www.dji.com/ground-station-pro). Flights were conducted around local noon time. The images were taken from a nadir view with 85% frontal and 80% side overlap.

The image pixel value depends on different factors, such as sensor setting, sensor properties, and scene condition, which must be corrected to get a radiometrically trusted measure of terrain reflectance. All related parameters are present in the image EXIF data and applied for radiometric corrections in Pix4D (refer to Shafiee et al., 2021, for more details). Reflectance targets were applied to do a radiometric calibration in field conditions. RedEdge-M has its calibration panel (with Albedo values of 0.58, 0.59, 0.60, 0.59, and 0.56, respectively, for the blue, green, red, red edge, and NIR bands). Our experiments showed that P4M generates more reliable spectral values when the radiometric calibration (data not shown) is applied, which was also noted by Di Gennaro et al. in their recent study (2022). Therefore, a calibration panel (SphereOptics, Diffuse Reflectance Target-53%R) was applied to correct P4M images (Albedo values of 0.54329, 0.54389, 0.54260, 0.54092, and 0.53788 respectively, for blue, green, red, red edge and NIR bands). Albedo values were determined for each central wavelength based on the datasheet the target provider enclosed. Images from both cameras were imported into Pix4D software (Pix4D SA, Lausanne, Switzerland) for processing. Different Processing steps of UAV images, including geometric correction, image mosaicking, and radiometric calibration, were conducted in Pix4D with a spatial resolution of 1.3 and 1.09 cm/pixel, respectively, for RedEdge-M and P4M. Orthomosaics were generated for each band separately. QGIS software (QGIS 3.4, Open-Source Geo-Spatial Foundation Project. http://qgis.osgeo.org) was used to extract average spectral values for each experimental plot in the trial (refer to Shafiee *et al*., 2021 for more details). Each plot was masked out in the middle part to avoid border effects using a polygon shape file in QGIS. A separate mask was generated for each camera. Since the pixel size is much smaller than the plot size and the canopy is well structured, the mixed pixel issue is insignificant in this study. The ZonalStatistic Tool was applied to calculate the median reflectance value per plot.

## Weather conditions during the field trials

Please refer to supplementary information of https://doi.org/10.1007/s00122-023-04424-9

## Planting dates of the trials

**Table S1** Planting dates of the field trials at both locations

| **Season** | **Vollebekk** | **Staur** |
| --- | --- | --- |
| 2015 | 2015-04-24 | - |
| 2016 | 2016-04-24 | 2016-05-10 |
| 2017 | 2017-05-04 | 2017-05-12 |
| 2019 | 2019-05-19 | 2019-06-04 |
| 2020 | 2020-05-15 | 2020-04-21 |
| 2021 | 2021-04-20 | 2021-04-27 |
| 2022 | 2021-04-21 | - |

## Band values during the season


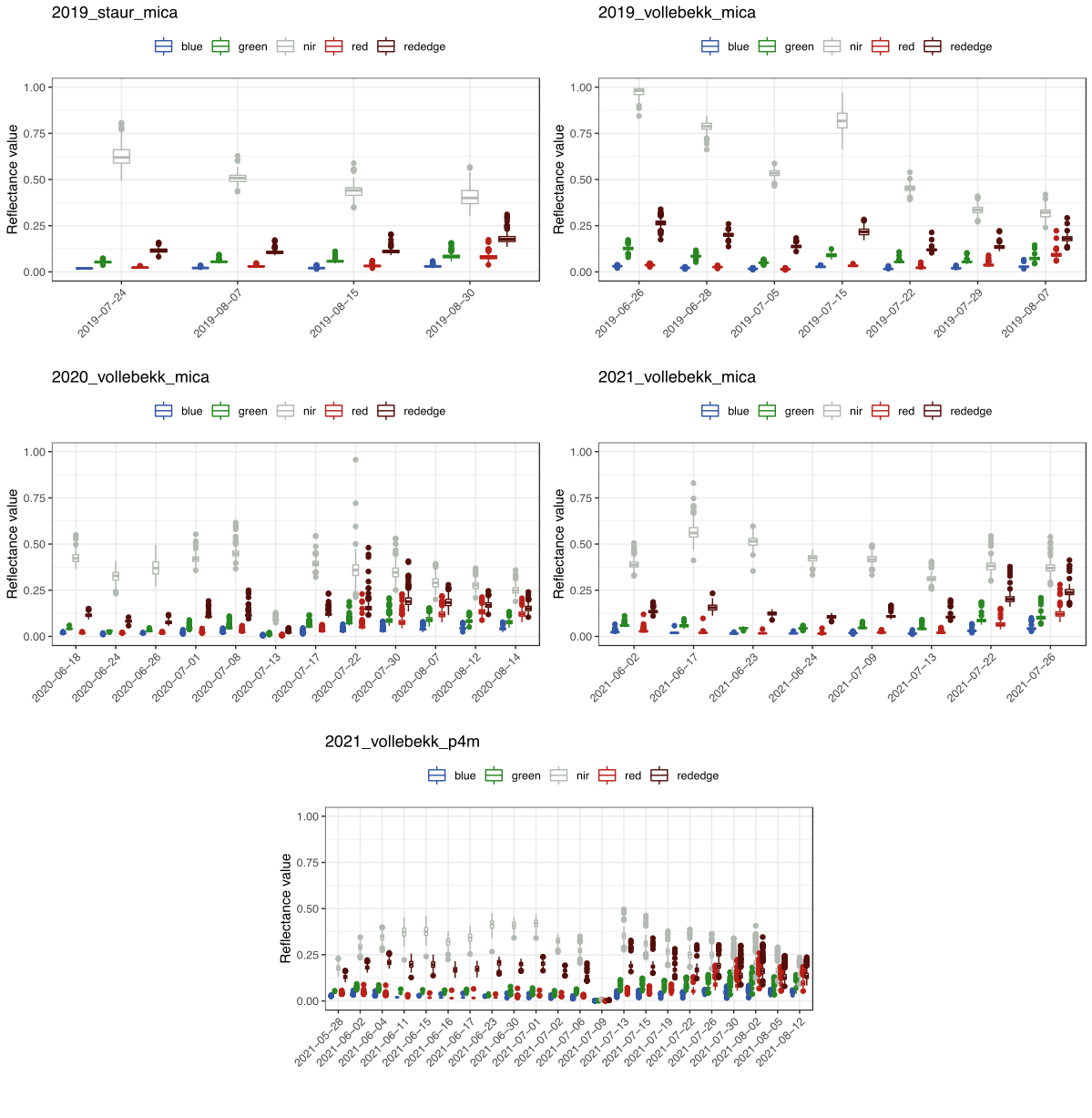


**Figure S1** Raw band reflectance during the season in all environments. For red, green, and blue, color on the chart represents the respective band; silver – NIR, dark red – RedEdge

## Correlations between the bands and grain yield


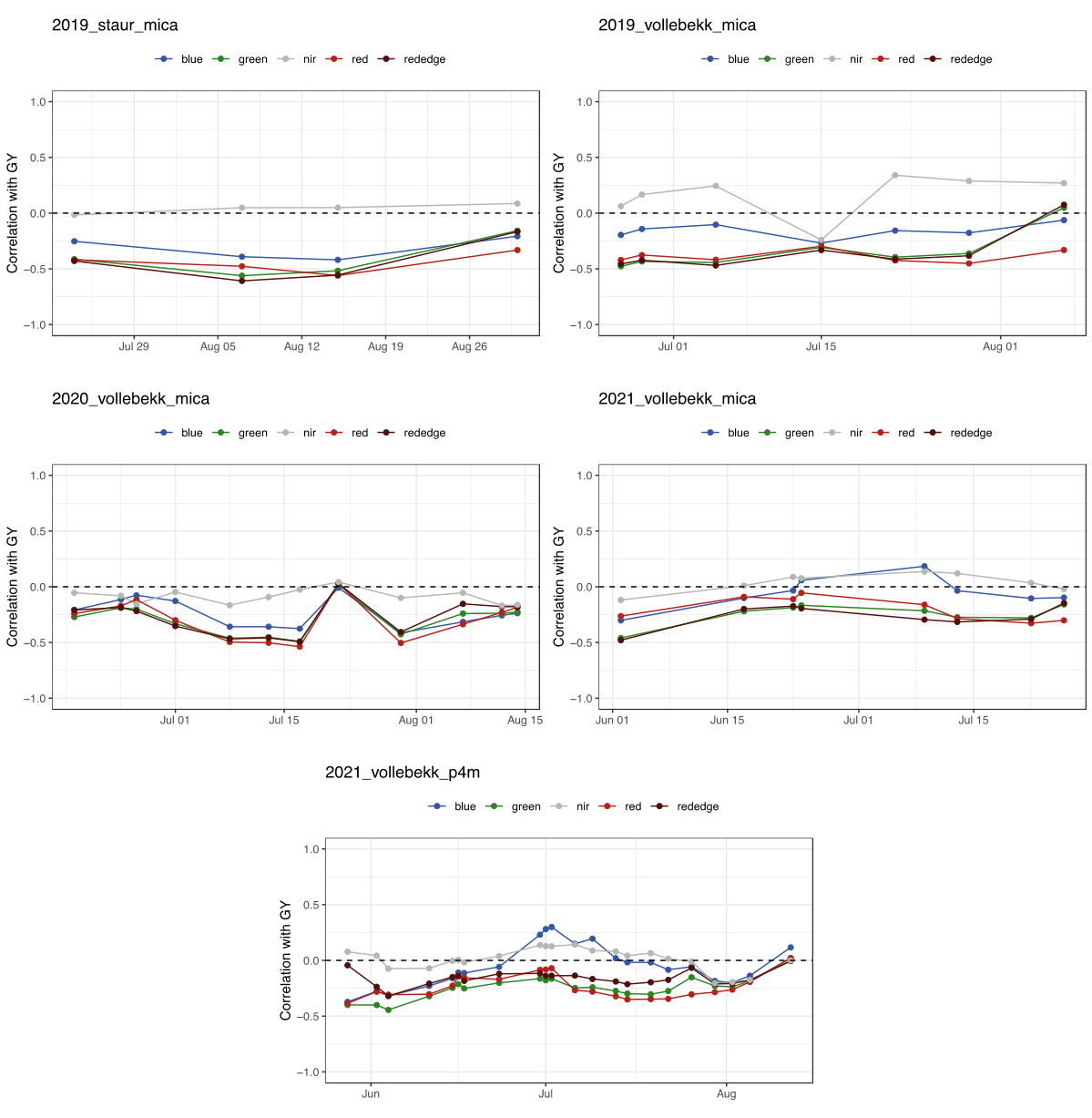


**Figure S2** Pearson correlation between the camera bands and grain yield for all the flight days. For red, green, and blue, the color on the chart represents the respective band; silver – NIR, dark red – RedEdge


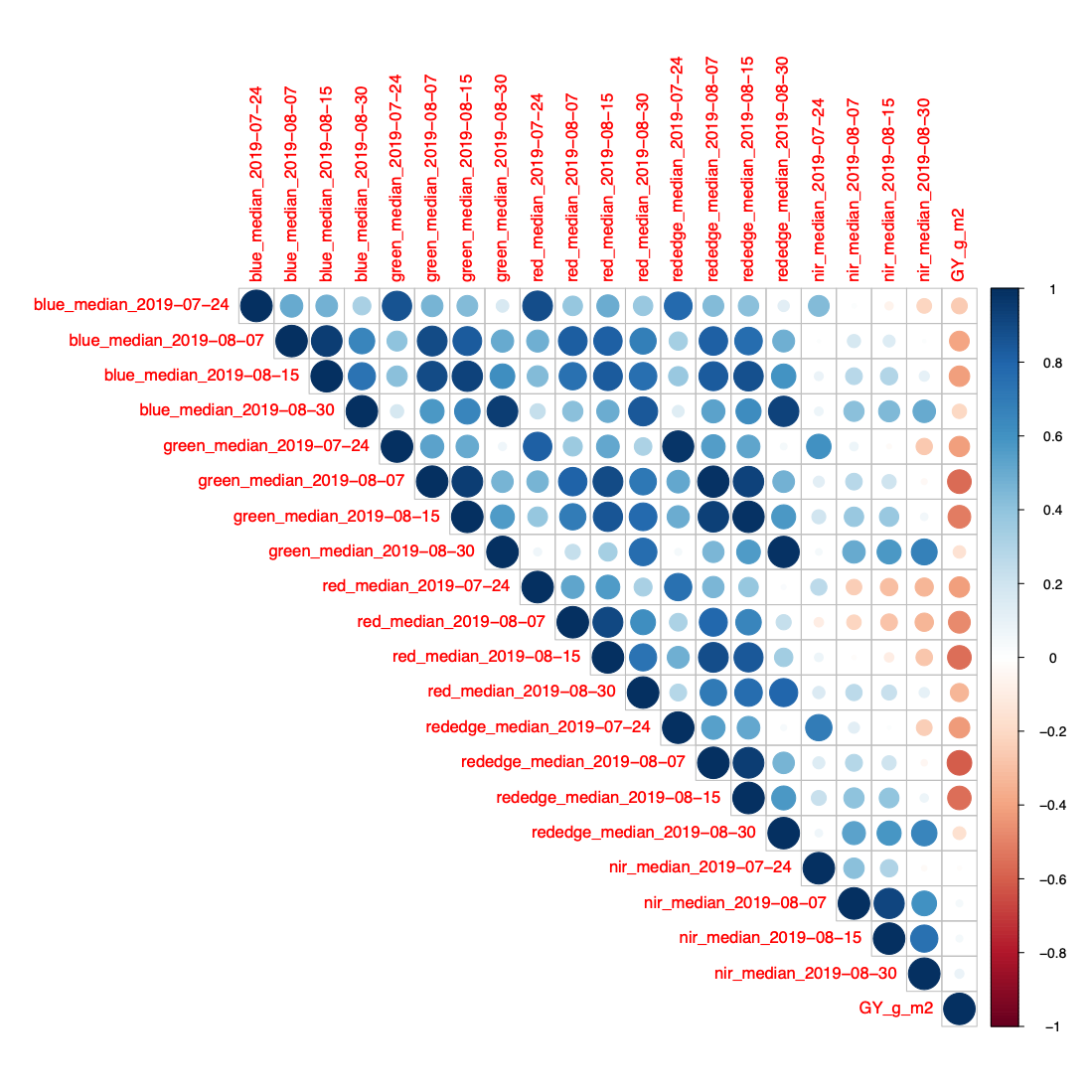


**Figure S3** Env: Staur 2019, Camera: Micasense. Pearson correlations among the bands at different days and grain yield


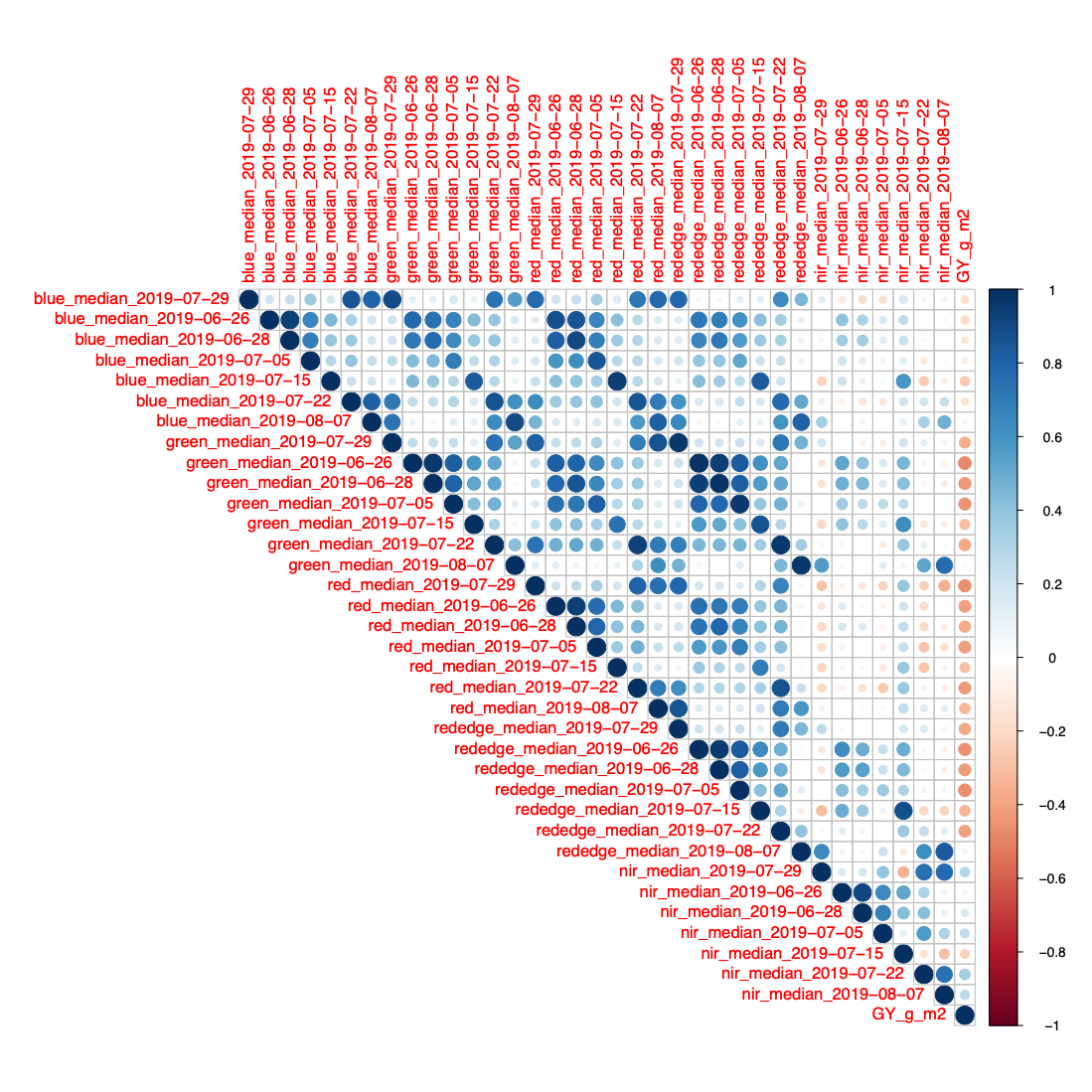


**Figure S4** Env: Vollebekk 2019, Camera: Micasense. Pearson correlations among the bands at different days and grain yield


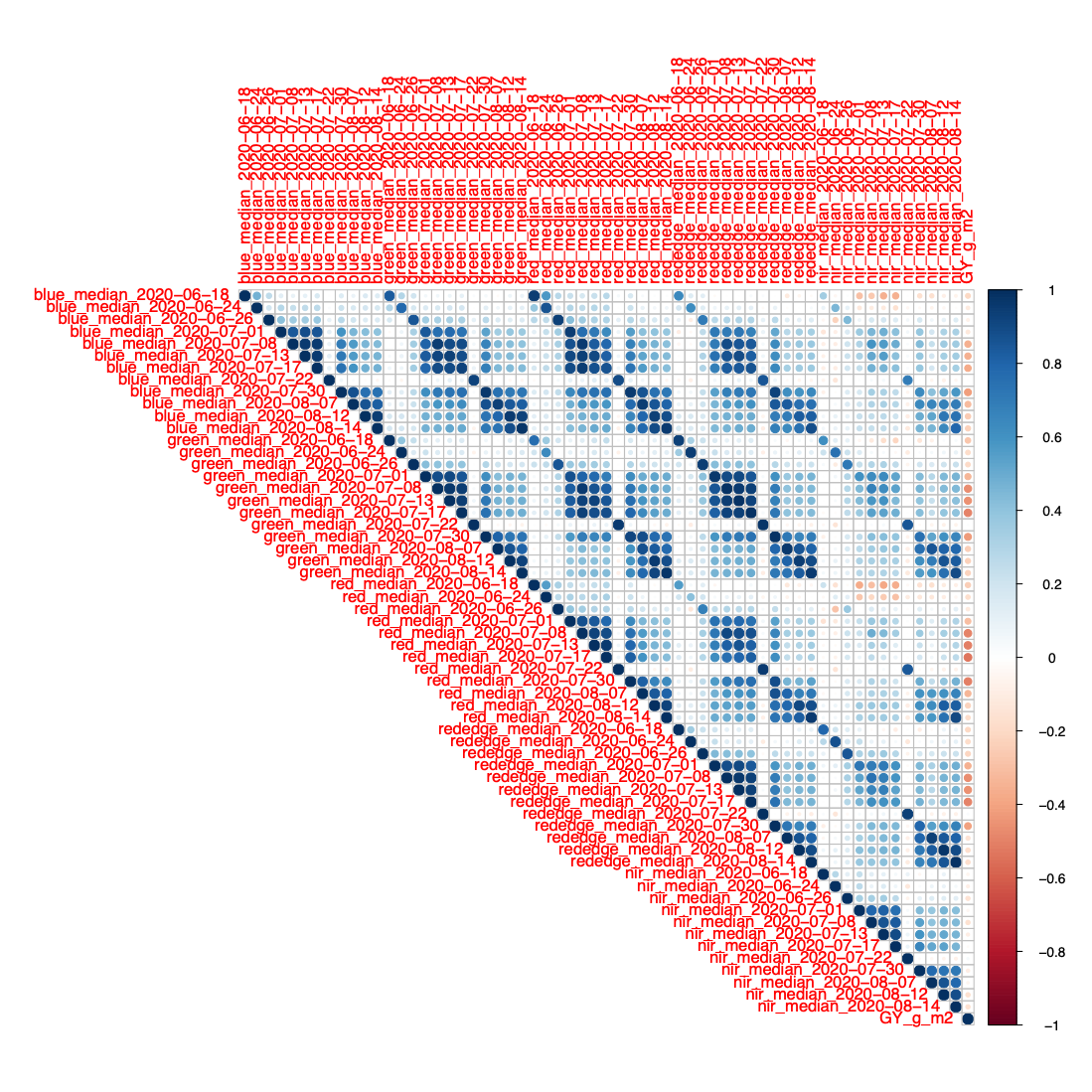


**Figure S5** Env: Vollebekk 2020, Camera: Micasense. Pearson correlations among the bands at different days and grain yield


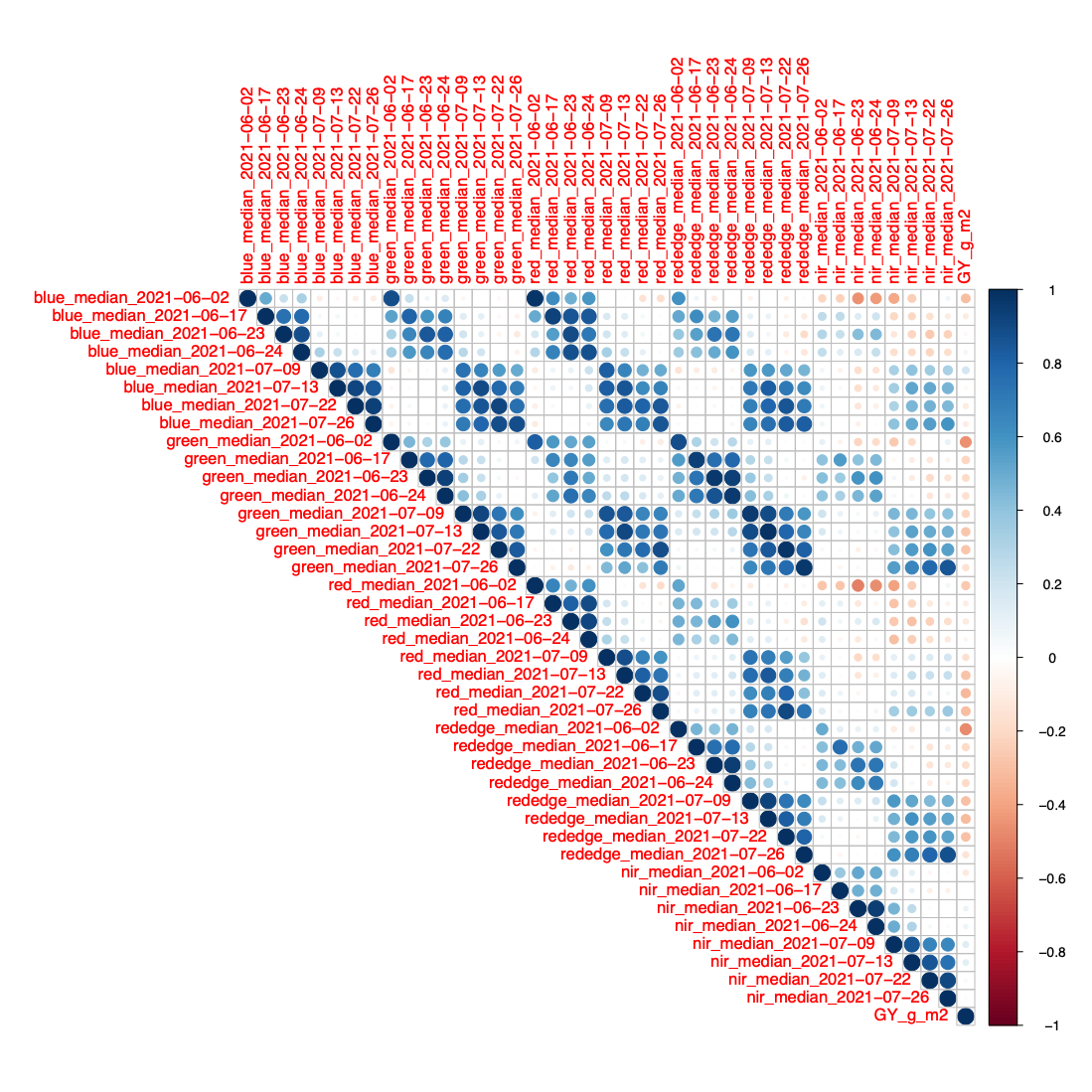


**Figure S6** Env: Vollebekk 2021, Camera: Micasense. Pearson correlations among the bands at different days and grain yield


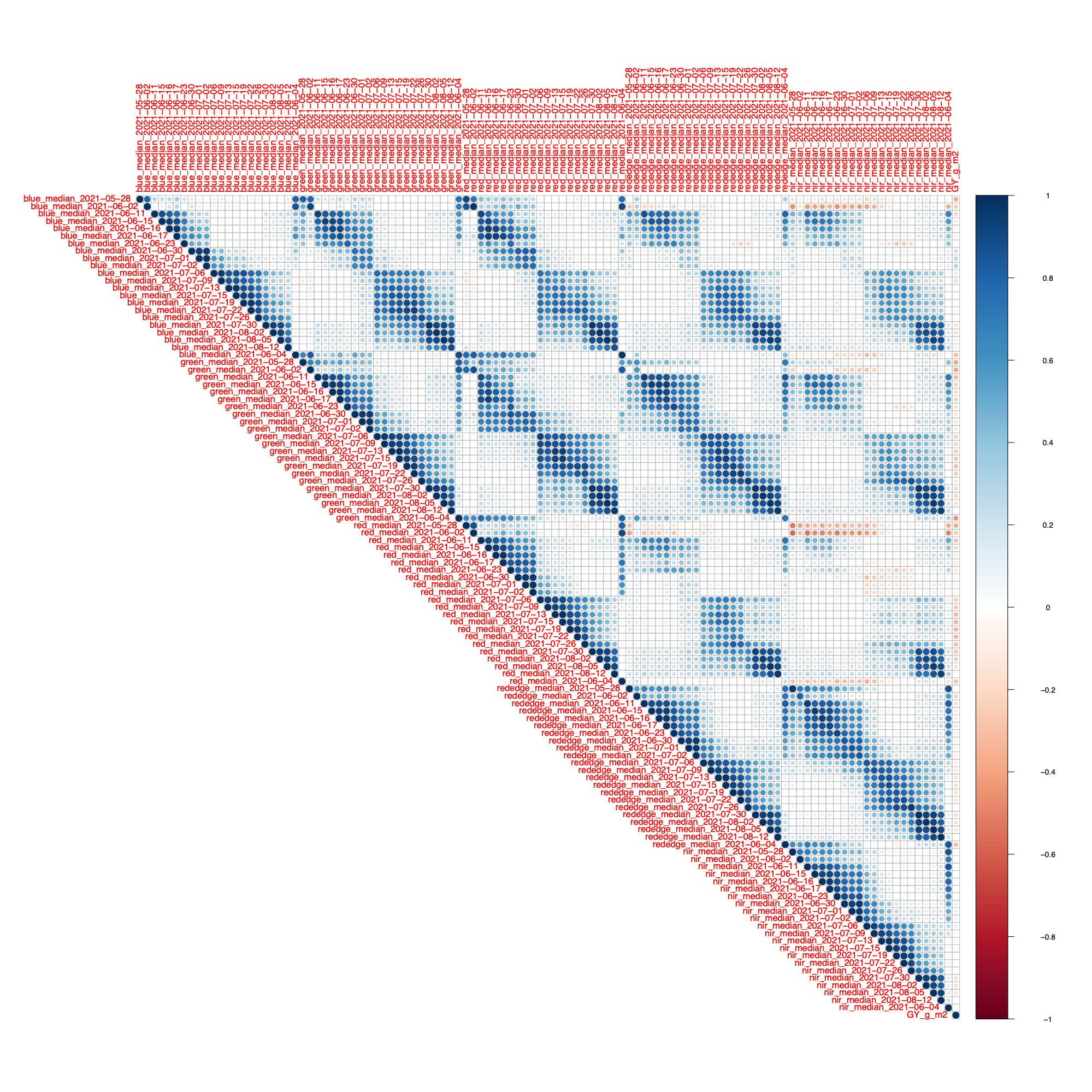


**Figure S7** Env: Vollebekk 2021, Camera: P4M. Pearson correlations among the bands at different days and grain yield
